# Hydrogen Peroxide Alters Splicing of Soluble Guanylyl Cyclase and Selectively Modulates Expression of Splicing Regulators in Human Cancer Cells

**DOI:** 10.1371/journal.pone.0041099

**Published:** 2012-07-20

**Authors:** Gilbert J. Cote, Wen Zhu, Anthony Thomas, Emil Martin, Ferid Murad, Iraida G. Sharina

**Affiliations:** 1 Department of Internal Medicine/Cardiology, University of Texas Medical School, UTHealth, Houston, Texas, United States of America; 2 Department of Endocrine Neoplasia and Hormonal Disorders, MD Anderson Cancer Center, Houston, Texas, United States of America; 3 Department of Biochemistry and Molecular Biology, George Washington University, Washington, DC, United States of America; University of Illinois at Chicago, United States of America

## Abstract

**Background:**

Soluble guanylyl cyclase (sGC) plays a central role in nitric oxide (NO)-mediated signal transduction in the cardiovascular, nervous and gastrointestinal systems. Alternative RNA splicing has emerged as a potential mechanism to modulate sGC expression and activity. C-α1 sGC is an alternative splice form that is resistant to oxidation-induced protein degradation and demonstrates preferential subcellular distribution to the oxidized environment of endoplasmic reticulum (ER).

**Methodology/Principal Findings:**

Here we report that splicing of C-α1 sGC can be modulated by H_2_O_2_ treatment in BE2 neuroblastoma and MDA-MD-468 adenocarcinoma human cells. In addition, we show that the H_2_O_2_ treatment of MDA-MD-468 cells selectively decreases protein levels of PTBP1 and hnRNP A2/B1 splice factors identified as potential α1 gene splicing regulators by *in silico* analysis. We further demonstrate that down-regulation of PTBP1 by H_2_O_2_ occurs at the protein level with variable regulation observed in different breast cancer cells.

**Conclusions/Significance:**

Our data demonstrate that H_2_O_2_ regulates RNA splicing to induce expression of the oxidation-resistant C-α1 sGC subunit. We also report that H_2_O_2_ treatment selectively alters the expression of key splicing regulators. This process might play an important role in regulation of cellular adaptation to conditions of oxidative stress.

## Introduction

Alternative splicing expands transcriptome diversity [1], [2] and allows cells to meet the requirements of an ever-changing extracellular environment. Common stressors such as heat-shock, amino acid starvation or ethanol toxicity have been demonstrated to regulate alternative splicing [3], [4]. Oxidative stress often persists in cellular microenvironment when there is an imbalance in the production and elimination of reactive oxygen species (ROS). This imbalance is associated with a plethora of pathologic conditions including carcinogenesis, cardiovascular disorders and neurodegeneration [5], [6], [7], [8], [9]. Recent evidence indicates that the alternative splicing can be influenced by changes in oxidative balance. Hypoxic and hypoxia/reoxygenation conditions, which alter ROS homeostasis, have been demonstrated to modify splicing of a number of genes in normal tissues and cancer cell lines [10], [11], [12], [13], [14]. Moreover, emerging data indicate that ROS can also alter the abundance of splicing factors or modify their activity. Low concentrations of hydrogen peroxide (H_2_O_2_), an ubiquitous ROS molecule, have been demonstrated to induce phosphorylation of the splicing factor hnRNP C leading to modulation of its RNA-binding affinity [15]. Increased hnRNP-C expression is also found in intimal hyperplasia and atherosclerosis, which is proposed to be associated with increases in H_2_O_2_ levels produced by activated vascular endothelium [16].

Soluble guanylyl cyclase (sGC) is a key enzyme of the nitric oxide (NO) signaling pathway and plays an important role in cardiovascular, neuronal and gastrointestinal functions [17]. Active sGC protein is a ferrous heme-containing heterodimer composed of α and β subunits, which is activated in response to the binding of NO to its heme moiety. Heme oxidation is proposed to be one of the mechanisms responsible for attenuation of NO/cGMP signaling in conditions of oxidative stress [18]. The sGC-specific inhibitor 1H-[1], [2], [4]oxadiazolo[4,3,-a]quinoxalin-1-one (ODQ) oxidizes sGC ferrous heme to the ferric form to suppress the binding of NO. Exposure of cells and tissues to ODQ triggers sGC degradation due to destabilization of sGC heterodimer through ubiquitination and targeted proteasomal degradation [18]. We have previously identified a novel alternatively spliced isoform of sGC, C-α1, which is fully functional despite an extensive deletion in N-terminus [19]. Surprisingly, C-α1 splice form was significantly more resistant to protein degradation induced by the treatment with ODQ [19] and had a different cellular localization in differentiating stem cells than the canonical α1 sGC [20]. Recent studies by Kraehling et al. revealed that, unlike the canonical α1 sGC isoform, the C-α1 isoform tends to localize to the more oxidized environment of the endoplasmic reticulum (ER) inside the cell [21]. These observations have led us to propose that the expression of the alternative C-α1 protein isoform can be induced by oxidative stress as a part of adaptation mechanism to preserve sGC activity in oxidative conditions.

In present study, we examined if splicing of α1 sGC gene can be modulated by ROS, specifically, by the treatment with H_2_O_2_. We found that H_2_O_2_ induces the expression of C-α1 transcript and C-α1 protein in human cancer cells expressing α1/β1 sGC. In an effort to gain insight into the regulatory mechanism, we examined the expression levels of several RNA binding proteins potentially involved in the splicing of C-α1 splice variant. Our studies show that H_2_O_2_ treatment selectively decreases the expression of putative α1 sGC splicing regulators PTBP1 and hnRNP A2/B1. Furthermore, we demonstrate that H_2_O_2_-induced degradation of PTBP1 differs in various breast cancer cell lines. To our knowledge, this is a first report demonstrating that H_2_O_2_-induced oxidative stress affects alternative splicing of sGC and selectively modulates protein level of major splice factors.

## Results

### H_2_O_2_ induces expression of C-α1 sGC splice variant

**Figure 1:**
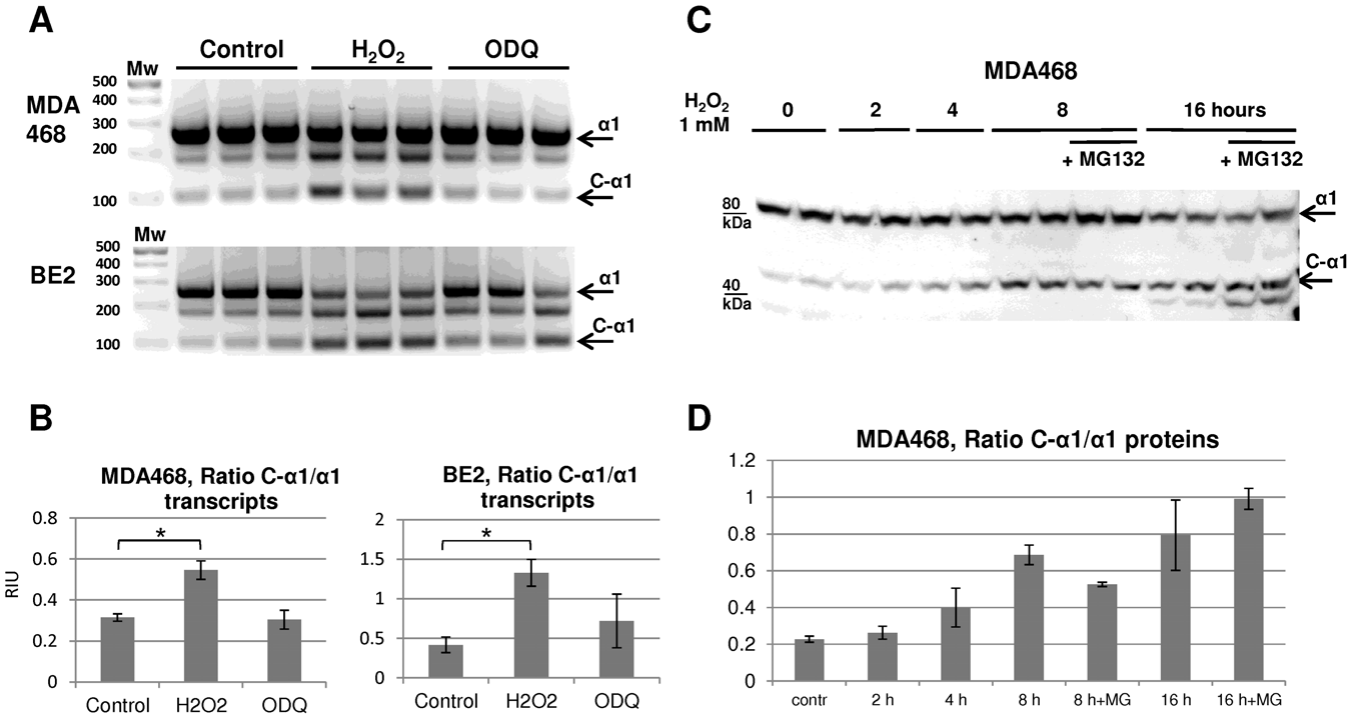
H_2_O_2_ exposure induces the expression of oxidation-resistant C-α1 sGC splice form in MDA468 and BE2 cells. A : RT-PCR detection of α1 (top) and C-α1 (bottom band) sGC transcripts following treatment with H_2_O_2_ (1 mM) or ODQ (20 µM), as indicated. RT-PCR products are separated on 3% agarose gel and stained with Ethidium Bromide. Upper band represents the message encoding canonical α1 protein (Transcripts 1–4, 270 bp); middle band is a non-specific product; bottom band indicates the C-α1 sGC transcript (Transcript 5, 94 bp). Biological triplicates representative of three independent experiments are shown. **B**: Ratio (mean ± SD) of the relative abundance of C-α1 and α1 transcripts quantified by densitometry. *p<0.05 by Student's t-test. **C**: Western blot detection of α1 (top) and C-α1 (bottom) proteins. MDA468 cells were treated with 1 mM H_2_O_2_ as indicated in presence or absence of MG132 (10 µM). Shown blots are representative of four independent experiments with similar results. **D**: Ratio of the relative abundance of C-α1 and α1 proteins quantified by densitometry. Data are shown as mean ± SD from four independent experiments.

To investigate if alternative splicing of GUCY1A3 (α1 sGC subunit gene) is regulated in response to oxidative stress, we treated human breast carcinoma MDA468 and human neuroblastoma BE2 cell lines with 1 mM H_2_O_2_. MDA468 and BE2 cells endogenously express α1 and β1 sGC. The H_2_O_2_ concentration was chosen based on previous observations demonstrating that interaction with serum proteins in cell culture media, absorption by cellular membranes and neutralization by enzymatic components of cellular anti-oxidative defense consume a significant portion of exogenously added H_2_O_2_ [22], [23]. MDA468 and BE2 cells demonstrated a significant increase in C-α1 sGC alternative transcript expression upon H_2_O_2_ treatment (Fig. 1 A, B). The relative increase of C-α1 isoform in comparison to α1 transcript was greater in BE2 cells, consistent with our previous studies [19]. The results indicated that GUCY1A3 splicing is altered to allow the recognition of C-α1 specific 3′ splice site within exon 4 in response to H_2_O_2_ exposure. Although in some experiments the level of C-α1 was increased by ODQ, neither cell line demonstrated statistically significant changes in C-α1 transcript level suggesting that ODQ treatment alone is not sufficient to affect splicing regulation.

Next, we evaluated the time-course of the changes in abundance of α1 and C-α1 sGC proteins in response to H_2_O_2_. Coincident with increases in GUCY1A3 splicing we observed a time-dependent modulation of α1 and C-α1 sGC protein levels. As shown in Fig. 1C and D, H_2_O_2_ increased the relative content of C-α1 protein in MDA468 cells. To assess the contribution of proteasome-dependent degradation in regulation of the α1 sGC splice forms, we performed the experiment in presence of MG132, a potent cell-permeable proteasome inhibitor. We found that pre-treatment with MG132 did not affect the rate of accumulation of C-α1 splice variant, or the level of the canonical α1 sGC subunit. These data suggest that the observed increase in C-α1 protein abundance (Fig. 1D) is likely due to an augmented C-α1 expression, and not to a selective degradation of canonical α1 sGC. Of note is that MG132 treatment also elevated levels of an unknown polypeptide recognized by anti-α1 antibodies with molecular weight lower than C-α1 sGC (Fig. 1D). As the identity of this protein remains to be determined, its intensity was not included in the densitometry analysis.

Together our results suggested that H_2_O_2_ induces preferential splicing and expression of C-α1 sGC splice form in our cell models.

### *In silico* analysis identifies potential α1 sGC (GUCY1A3) splicing regulators

**Figure 2:**
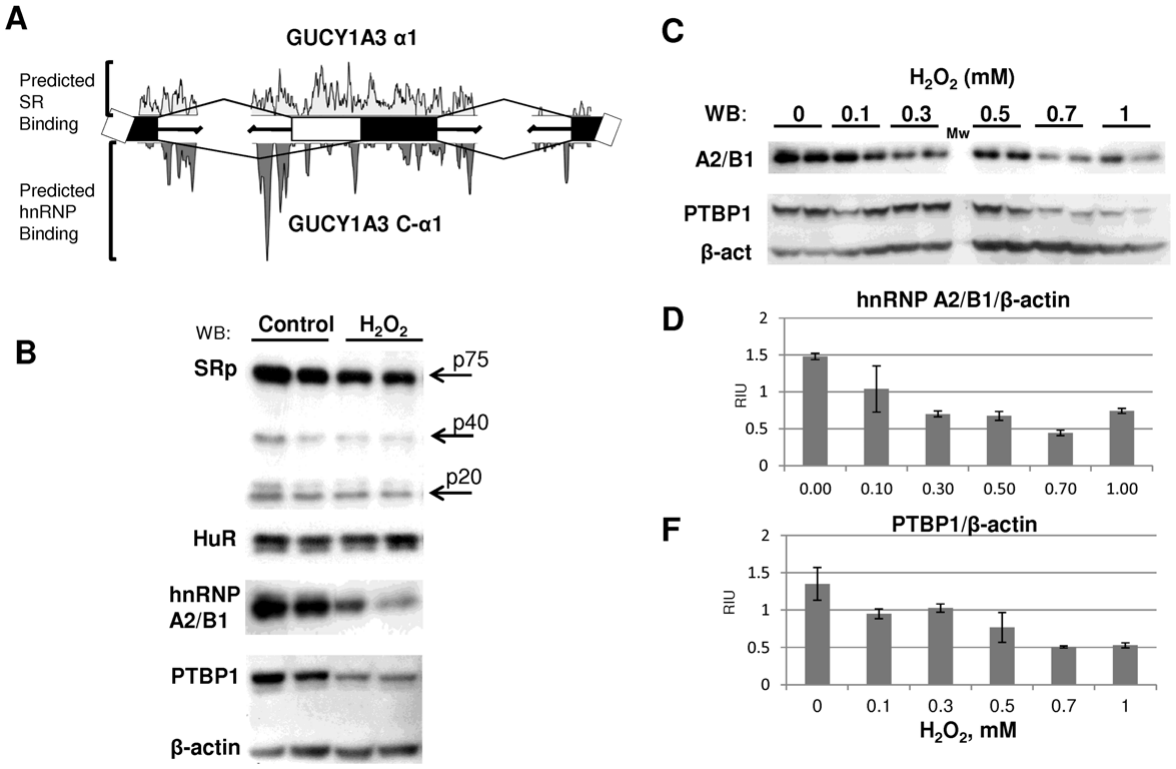
H_2_O_2_ exposure selectively alters the expression of splice factors predicted to regulate sGC splicing in MDA468 cells. **A**: Schematic representation of GUCY1A3 alternative splicing to generate C-α1. The relative distribution of predicted SR and hnRNP binding sites is shown. **B**: Western analysis of PTBP1, hnRNP2A/1B, HuR and SR proteins expression in control and H_2_O_2_-treated (1 mM, 24 hours) MDA468 cells. **C**: H_2_O_2_ dose-dependent assessment of PTBP1 and hnRNP A2/B1 protein levels in MDA468 cells. Shown Western blots are representative of at least three independent experiments with similar results. **D, F**: Densitometry analysis of PTBP1 and hnRNP A2/B1 protein levels normalized on β-actin. Data are shown as mean ±SD from three independent experiments.

To gain the initial insight into potential mechanisms of α1 sGC splicing regulation, we performed *in silico* analysis using several bioinformatics tools [24]. The C-α1 splice variant is generated by the use of an alternative 3′ splice site 179 base pairs downstream of the constitutive site (Fig. 2A, Fig. S1). Constitutive and alternative splice sites for exon 4 of α1 sGC were defined with the UCSC Genome Bioinformatics tool and their relative consensus value examined using Human Splicing Finder [25], [26]. Alternative splicing of exon 4 plays a central role in GUCY1A3 transcript diversity; in addition to the constitutive site, the exon contains 2 alternative donor sites and 2 alternative acceptor sites (see Fig. 2A and Fig. S1). With the exception of the constitutive 5′ splice site donor, the relative consensus strength of all sites proved to be low (Fig. S1). This is a common feature of alternatively processed exons that is thought to facilitate regulation by the serine-arginine rich (SR) and heterogeneous nuclear ribonucleoprotein (hnRNP) proteins [2]. The ASD, Alternative Splicing/Splicing Rainbow tool from European Molecular Biology Laboratory [27], was used to analyze the relevant exon and proximal intron sequences for potential SR and hnRNP regulators of exon 4 splicing. We generated a composite overview of regulator binding sites using the derived values for individual SR and hnRNP proteins (Table S1). This analysis identified a relatively even distribution of SR binding sites throughout exon 4 and the flanking regions. At the same time, the constitutive 3′ splice site intron region showed a dense peak of hnRNP binding sites (Fig. 2A). A closer examination identified in this sequence several overlapping binding sites for hnRNP 2A/B1, polypyrimidine-tract-binding protein 1 (hnRNP I, PTBP1), SRp20, SRp40 and Hu antigen R (HuR, ELAVL1) splicing regulators (Fig. S1). The location of these sites suggested that corresponding splice factors are likely to affect the use of both canonical and alternative splice sites, promoting the generation of the C-α1 sGC transcript.

### H_2_O_2_ selectively alters the expression of splicing factors

It is well established that the expression levels and the relative stoichiometry of splice factors modulate alternative splicing [28]. Therefore, we investigated the effect of H_2_O_2_ exposure on protein levels of the splice factors identified by our *in silico* analysis as potential sGC regulators. As shown in Fig. 2B, the exposure of MDA468 cell to H_2_O_2_ selectively decreased the protein level of PTBP1 and hnRNP A2/B1 splicing repressors. H_2_O_2_ did not affect the level of HuR regulator and only slightly altered levels of SRp40 protein from SR family of splicing enhancers. Both PTBP1 and hnRNP A2/B1 proteins are decreased in a dose-dependent manner in MDA468 cells (Fig. 2 C, D and F). Our data indicate that the observed H_2_O_2_-dependent switch in splicing of GUCY1A3 gene coincides with the changes in the level of regulatory splice factors. However, the exact mechanism and the contribution of specific splice factors in the regulation of sGC splicing remains to be determined.

### Insights into the mechanism of H_2_O_2_-induced PTBP1 protein degradation

Next we explored potential mechanisms of hnRNP regulation by H_2_O_2_. We chose to focus on PTBP1 since this extensively studied RNA-binding protein plays an important role in various steps of cellular mRNA processing, including splicing, regulation of stability, localization and translation [29]. Moreover, PTBP1 has been demonstrated to be essential in the regulation of cell growth and cancer cells survival [30], [31], [32]. The ability of H_2_O_2_ to reduce PTBP1 levels suggested that, additionally, it may play a role in the regulation of cellular adaptation to oxidative conditions. However, the effect of elevated ROS levels on PTBP1 expression has never been examined previously.

**Figure 3:**
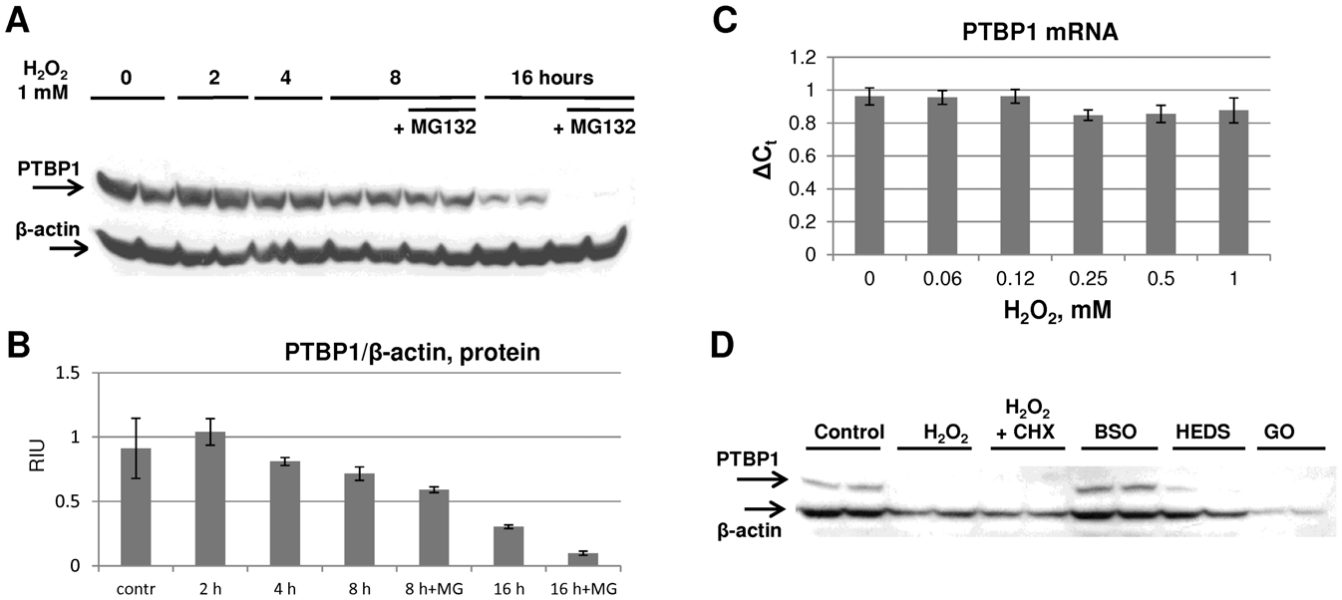
Insights into the mechanism of H_2_O_2_-induced PTBP1 down-regulation. **A**: PTBP1 degradation occurs in time-dependent manner and is not prevented by proteasome inhibitor MG132. MDA468 cells were treated as in Fig. 1C with 1 mM H_2_O_2_ in the presence or absence of MG132 (10 µM). Western blot analysis was performed to visualize the expression of PTBP1. β-actin served as a loading control. Biological duplicates for each treatment are shown; blots are representative of three independent experiments with similar results. **B**: Densitometry analysis of PTBP1 protein levels normalized on β-actin. Averages for representative biological duplicates for each treatment are shown. **C**: H_2_O_2_ exposure does not affect PTBP1 mRNA levels. MDA468 cells were treated with indicated concentrations of H_2_O_2_ for 24 hours. Relative abundance of PTBP1 mRNA in samples was analyzed by RT-qPCR analysis. Average ΔCt ± SD for biological triplicates are shown. **D**: PTBP1 degradation depends on thiol oxidation and is not rescued by inhibition of *de novo* protein synthesis. Western blot analysis performed on MDA468 cell lysates treated for 24 hours with inhibitor of protein synthesis cycloxemide (2 µg/ml) and different factors inducing oxidative stress: 1 mM BSO (GSH depletion inducer); 1 mM HEDS (thiol oxidation inducer) and 0.01 units/ml of Glucose Oxidase (increases production of ROS). Shown Western blots are representative of three independent experiments with similar results.

We first investigated if H_2_O_2_ alters PTBP1 mRNA steady-state levels. RT-qPCR analysis performed with RNA samples isolated from MDA468 cells treated with different H_2_O_2_ concentrations found no changes in PTBP1 mRNA levels (Fig. 3C) indicating that PTBP1 is likely to be controlled at the protein level. To examine the role of proteasomal degradation, we monitored the dynamics of changes in PTBP1 protein in response to H_2_O_2_ treatment over a 16 hours period in the presence or absence of proteasome inhibitor MG132. We found that PTBP1 protein was reduced in a time-dependent manner starting at 8 hours post-exposure (Fig. 3 A, B). Interestingly, the protein degradation was not prevented, but rather enhanced, by MG132 treatment. This was particularly evident at the 16-hour and later time points (Fig. 3A and data not shown). Thus, while MG132 treatment showed that the proteasome had no direct effect on PTBP1 degradation, the fact that its inhibition accelerates degradation implies an indirect role in regulation, most likely through stabilization of an unknown protease or protein cofactor. The pre-treatment of MDA468 cells with cycloheximide (CHX, the inhibitor of *de novo* protein synthesis) also did not prevent PTBP1 decrease, indicating that H_2_O_2_ was inducing a pre-existing protein degradation pathway (Fig. 3D). It has been previously demonstrated that cellular apoptosis leads to PTBP1 degradation through Capase-3 activation [33]. Thus, we explored the possibility that Caspase-3 might play a role in H_2_O_2_-induced PTBP1 decreases. However, we found that the addition of Caspase-3 inhibitor IV (Ac-DMQD-CHO, Calbiochem) did not prevent H_2_O_2_–induced PTBP1 degradation (results are not shown), indicating that Caspase-3 is not involved in this process. To determine whether other exogenous sources, besides direct addition of H_2_O_2_ solution, might affect the stability of PTB1, we applied glucose oxidase (GO) to the cell culture media. Consistent with the direct role of H_2_O_2_ in the induction of PTBP1 degradation, steady-state production of H_2_O_2_ by GO treatment reproduced the effect.

H_2_O_2_ induces post-translational protein modifications such as oxidation of intracellular thiols and thiolate anions. These modifications are an integral part of H_2_O_2_ signaling affecting a variety of cellular processes [34]. It has been previously demonstrated that in oxidative conditions PTBP1 may form dimers due to the formation of intermolecular disulfide bridges [35]. Indeed, in our experiments we also detected the formation of a high molecular weight protein band recognized by PTB1 antibodies following the treatment of MDA468 cells with H_2_O_2_ (results are not shown).

To evaluate the contribution of thiol oxidation to PTBP1 degradation, we exposed MDA468 cells to two different compounds altering cellular thiol metabolism. Treatment with 2-hydroxyethyl disulphide (HEDS), an agent that acts as thiol-specific oxidant, significantly decreased PTBP1 levels (Fig. 3D), indicating that direct thiol oxidation might play an important role in PTBP1 degradation response. To further test if PTBP1 degradation is induced by diminished cellular thiol-reducing activity in response to H_2_O_2_, we also treated the cells with L-buthionine-S,R-sulfoximine (BSO). BSO is an irreversible inhibitor of glutathione biosynthesis, decreasing intracellular glutathione pool. We observed no significant degradation of PTBP1 in response to BSO, suggesting that a decreased level of GSH is not sufficient to induce PTBP1 (Fig. 3D) degradation. These results may also be related to the intrinsic ability of MDA468 cells to compensate for the GSH loss induced by BSO, as has been previously reported for cell lines with high SOD expression [36]. Thus, oxidation of Cys thiols by H_2_O_2_ could initiate the formation of the PTBP1 dimers and contribute to subsequent protein degradation.

### H_2_O_2_ effect on PTBP1 protein levels varies in different breast cancer cell lines

**Figure 4:**
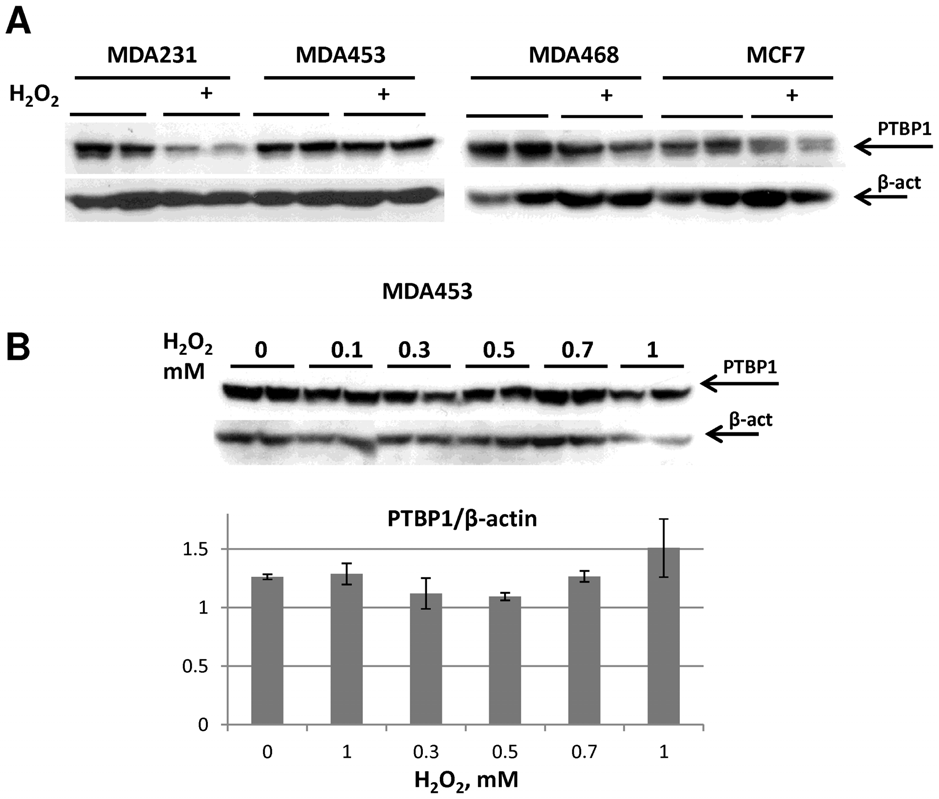
PTBP1 response to H_2_O_2_-induced degradation varies in different breast cancer cell lines. **A**: Western blot analysis examining PTBP1 expression in MDA231, MDA453, MDA468 and MCF7 cells treated with 1 mM H_2_O_2_ for 18 hours. Representative biological duplicates are shown. **B**: MDA453 cells are resistant to H_2_O_2_-induced PTBP1 degradation. *Top panel*: MDA453 cell were treated with different concentration of H_2_O_2_ and cell lysates were subjected to Western blot analysis with antibodies towards PTBP1 and β-actin. Shown blots are representative of four independent experiments with similar results. *Bottom panel*: densitometry analysis of PTBP1 protein levels normalized on β-actin levels. Averages for representative biological duplicates for each treatment are shown.

**Figure 5:**
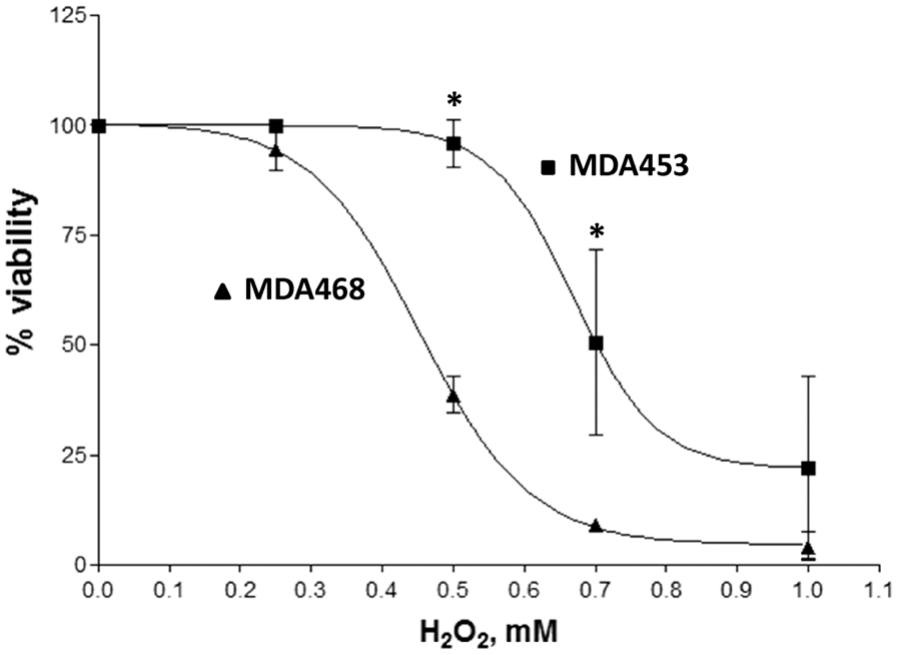
H_2_O_2_ cytotoxicity analysis in MDA468 and MDA453 cells. Survival curve was generated in response to H_2_O_2_ dosage using trypan exclusion method and expressed as % of survival to untreated controls. Mean ± SD of three independent passages performed in triplicates are shown, *- p<0.05 by Student's t-test in comparison to control.

To determine the generality of H_2_O_2_-induced PTBP1 degradation, we tested additional breast cancer lines, including: MDA-MD-453, MDA-MD-231 and MCF7. Western blot analysis of H_2_O_2_-treated cells demonstrated various degrees of PTBP1 reduction in MDA468, MCF7 and MDA231 cells, but no change in MDA453 cells (Fig. 4A). The failure to induce PTBP1 degradation in MDA453 cells was observed at H_2_O_2_ concentrations that readily elicited significant decreases in MDA468 cells (compare Fig. 4B to Fig. 2B and C). These data suggested that the extent of H_2_O_2_-induced degradation of PTBP1, thought prevalent, is still rather cell line specific. To explore if the resistance to PTBP1 protein degradation is associated with increased ability of cells to survive oxidative stress, we compared H_2_O_2_-induced cytotoxicity in MDA468 and MDA453 cells. MDA468 cells were less resistant to H_2_O_2_-induced cytotoxicity (IC_50_ = 450±60 µM) compared with MDA453 cells (IC_50_ = 660±2 µM) (Fig. 5). In an attempt to directly link a reduction in PTBP1 levels to H_2_O_2_-induced cytotoxicity we performed siRNA-mediated PTBP1 knockdown in MDA453 cells. Despite a greater than 50% reduction in PTBP1 mRNA (as detected by RT-qPCR), cell viability measurements revealed only a small insignificant decline in resistance to H_2_O_2_ treatment (Fig. S2). These data suggest that the level of PTBP1 expression alone is not critically important to support oxidative resistance in MDA435 cells.

## Discussion

In this report we demonstrate that the oxidative stress induced by H_2_O_2_ influences splicing of the α1 sGC gene (GUCY1A3) and selectively decreases protein level of PTBP1 and hnRNP A2/B1 splicing factors. We previously established that GUCY1A3 transcripts undergo alternative splicing and that the C-α1 sGC splice isoform encodes a protein that is resistant to ODQ-induced degradation [19], [37]. ODQ induces degradation through oxidation of the sGC prosthetic heme group, which destabilizes the protein. A similar process has been proposed to occur in conditions of oxidative stress and to be responsible for decreased level of sGC protein in vascular inflammation [23]. In this study we investigated if sGC splicing can be modulated by ROS as a potential mechanism to favor of the expression of oxidation resistant C-α1 splice form.

H_2_O_2_ is an ubiquitous ROS molecule that is produced in cells by superoxide dismutases as a metabolite of superoxide anion (O^2−^). H_2_O_2_ is a relatively stable and neutrally charged which enables it to readily cross cell membranes. These properties have led to the proposal that H_2_O_2_ may be involved in paracrine oxidative stress signaling [38], [39]. Thus, we chose to examine H_2_O_2_ as a mediator of oxidative stress in our studies performed on human BE2 neuroblastoma and MDA468 breast adenocarcinoma cell lines. Our data demonstrated for the first time that an exposure to H_2_O_2_ increases the relative amount of C-α1 mRNA and protein in these cell lines (Fig. 1). This result establishes that C-α1 splicing can be modulated by physiologically relevant ROS compound, and suggests that splicing may play an important role in modulating sGC enzymatic properties in response to changes in oxidative balance of the microenvironment. An additional study in support of this idea has recently been reported by Kraehling et al. [21]. This report independently confirmed our previous findings that C-α1 sGC forms highly stable and fully active heterodimers with β1 sGC . Furthermore, the difference in subcellular distribution of C-α1 and canonical α1 sGC subunits has been demonstrated using fluorescently tagged protein; the C-α1 isoform was localized to the more oxidized environment of endoplasmic reticulum. Together these data suggest that alternative splicing of GUCY1A3 gene might participate in adaptive cell response to preserve sGC function in oxidative stress. Further studies to evaluate the physiological importance of such adaptation are necessary.

To gain an insight into potential mechanisms involved in alternative splicing of α1 sGC, we performed an *in silico* analysis of relevant GUCY1A3 intronic and exonic sequences to identify potential regulatory elements [40], [41]. The distribution of predicted *cis*-regulatory sequences was consistent with a model whereby SR proteins would favor canonical splice site use, while binding of hnRNPs, specifically PTBP1 and hnRNP A2/B1, would block recognition of the exon 4 constitutive 3′ splice site to promote C-α1 isoform production (Fig. 2A and Fig. S1). To test our model we examined the expression of splice factors before and after exposure to H_2_O_2_. While splicing regulation is commonly achieved by modulating the expression level of regulatory factors [28], the impact of oxidative stress on this process has not been characterized previously. Contrary to our expectations, the data showed that H_2_O_2_ exposure induced a selective reduction of PTBP1 and hnRNP A2/B1, and had no effect on HuR and SR protein expression (Fig. 2). Our experimental results indicate a more complex regulation of sGC splicing than that predicted by *in silico* model. Further investigations are needed to explain this difference. On another hand, the specific and dramatic reduction of two key RNA splicing factors suggested that selective down-regulation of splice regulators could be one of the mechanisms underlying the changes in alternative splicing in response to oxidative stress.

PTBP1 plays a crucial role in a number of post-transcriptional regulatory processes, and its activity has been linked to cellular proliferation, apoptosis, tumorigenesis and response to hypoxia [30], [42], [43], [44]. Therefore, we chose to further explore the effect of H_2_O_2_ treatment on the PTBP1 expression. Our results demonstrated that H_2_O_2_ decreases PTBP1 expression in a dose- and time-dependent manner (Fig. 2C, F and Fig. 3A, B). No change in PTBP1 mRNA level was observed, indicating that the down-regulation occurs post-transcriptionally (Fig. 3C). Furthermore, the several hours delay in response to H_2_O_2_ treatment suggests an indirect mechanism of PTBP1 down-regulation. This conclusion is supported by the observation that proteasome inhibitor MG132 facilitated, and not inhibited, the H_2_O_2_-induced decline in PTBP1 protein (Fig. 3A, B). Because PTBP1 degradation did not require new protein synthesis (it was not blocked by cycloheximide treatment) our findings indicate that H_2_O_2_ may potentially enhance proteasomal degradation of some unknown factor responsible for PTBP1 stabilization. Given that H_2_O_2_ exposure is known to induce apoptosis [45], we considered a caspase-mediated degradation of PTBP1. Previous studies have demonstrated that PTBP1 is targeted by Capase-3 during apoptotic response [33]. However, Inhibitor IV failed to prevent H_2_O_2_-mediated reduction in PTBP1 protein levels (data not shown). Thus, we conclude that H_2_O_2_ probably induces PTBP1 degradation by a different from previously described mechanisms.

It is also important to point out that PTBP1 degradation was not limited to the direct addition of H_2_O_2_ solution to cells. Application of extracellular glucose oxidase (GO), which catalyzes the conversion of glucose into H_2_O_2_ and D-glucono-δ-lactone, also induced a significant decline of PTBP1 protein in MDA468 cells (Fig. 3D).

The reaction of H_2_O_2_ with protein thiols (R-SH) and thiolate anions (R-S^−^) is known to generate sulfenic acids (RSOH) modifications and promote disulfide bond formation. These proteins were implicated in a wide variety of biochemical effects mediating ROS signaling [34], [46]. Interestingly, dimerization of PTBP1 molecules via intermolecular Cys bridge formation was previously observed in oxidative conditions [35]. We explored the possibility that down-regulation of PTBP1 protein by H_2_O_2_ is mediated by thiol oxidation. Indeed, non-specific thiol oxidizing compound HEDS elicited a significant decline of PTBP1 levels, similar to a direct H_2_O_2_ exposure (Fig. 3D). Thus, dimerization through cysteine oxidation might target PTBP1 protein to subsequent degradation. Delineation of an exact mechanism responsible for selective ROS-induced degradation of this important regulator may offer additional important insights into cellular oxidative response.

Previously, PTBP1 expression has been correlated with increases in proliferation and metastatic potential of cancer cells; however, this effect varies in different cell lines [31], [32], [47], [48]. In addition, elevated PTBP1 levels have been shown to support aerobic glycolysis and enhance translation of hypoxia-inducible factor 1α (HIF-1α), which enables cancer cells to survive hypoxic conditions associated with altered ROS homeostasis [42], [49], [50], [51]. We employed several breast cancer lines to investigate if the effect of H_2_O_2_ on PTBP1 levels has a general nature. We observed that similar concentrations of H_2_O_2_-induced PTBP1 degradation in three out of four investigated breast cancer lines (Fig. 4A). The highest difference was observed between MDA468 and MDA453 lines. Similar range of H_2_O_2_ concentrations induced significant decreases in the level of PTBP1 in MDA468 cells, but did not affect PTBP1 level in MDA453 cells (compare Fig. 2C, D vs Fig. 4B). Thus, we can conclude that although the majority of breast cancer cell lines we tested (3 out of 4) responded to H_2_O_2_ treatment by down-regulation PTBP1 levels, the response is not universal. This raised the obvious question of whether a failure to degrade PTBP1 is associated with changes in cytotoxic response to H_2_O_2_. Indeed, we found that MDA453 cells were significantly less sensitive to cytotoxic concentrations of H_2_O_2_ than MDA468 (Fig. 5). This observation is in agreement with previous reports suggesting that preserving PTBP1 expression is beneficial to cancer cells survival in oxidative stress [30], [31]. However, we were unable to demonstrate a direct role for PTBP1 in MDA453 cells response to H_2_O_2_, as siRNA-mediated knockdown had no effect on cytotoxicity (Fig. S2). Additional studies are necessary to uncover the precise role of PTBP1 in H_2_O_2_–mediated cytotoxicity and determine if the lack of PTBP1 degradation in response to a treatment with oxidants may serve as a marker for resistance to oxidative stress in individual cancer cell lines.

In summary, our data demonstrate that the oxidative stress induced by H_2_O_2_ promotes splicing of oxidation-resistant C-α1 sGC splice variant and selectively alters protein levels of major splice factors.

## Materials and Methods

### Cell Culture and Preparation of Protein Lysates

BE2 human neuroblastoma cell line (American Type Culture Collection) was cultured in DMEM/F12K media supplemented with 10% FBS, 0.1 mM MEM nonessential amino acids, penicillin-streptomycin mixture (50 units/ml and 50 µg/ml), 10 mM Hepes (pH 7.4), 1 mM sodium pyruvate, 2 mM L-glutamine (all from Gibco/Invitrogen) and maintained at 37°C and 5% CO_2_. Human adenocarcinomas MDA-MD-468, MCF7, MDA-MD-231 and ductal carcinoma MDA-MD-453 cells (generous gift of Dr. Hesham Amin, MD Anderson Cancer Center) were cultured in RPMI supplemented with 10% FBS, 0.1 mM MEM nonessential amino acids and penicillin-streptomycin mixture (50 units/ml and 50 µg/ml) and maintained at 37°C and 5% CO_2_. For treatments, 70–80% confluent cell cultures were exposed with different agents up to 24 hours. To prepare lysates, the cells were collected by trypsinolysis, washed twice with PBS, re-suspended in 50 mM TEA (pH 7.4) containing protease inhibitor cocktail (Sigma-Aldrich, St. Louis, MO) and disrupted by sonication. The lysates were centrifuged at 15,000× g for 30 min to prepare the cleared supernatant fractions, which were used for Western blotting.

### Reverse Transcriptase-Polymerase Chain Reaction (RT-PCR)

Total RNA from cells was isolated using UltraSpec total RNA isolation reagent (Biotecx, Houston, TX). cDNA was prepared using a high-capacity cDNA kit (Applied Biosystems, Foster City, CA). The semi-quantitative RT-PCR assay for detection of C-α1 sGC mRNA (Transcript 5, NCBI, Accession N NM_001130685) was performed as described previously [19]. A set of specific primers flanking the deletion in α1 sGC Transcript 5 was used to perform PCR amplification. Primer sequences were as follows: Forward Primer 5′-cagccccgaggtgtgcgaag-3′; Reverse Primer 5′-ggcacggttgctttgcagct-3′. PCR products representing canonical α1 sGC mRNA (270 bp, encoding full size protein) and Transcript 5 mRNA (94 bp, encoding C-α1 sGC splice variant), were separated on agarose gel and visualized by Ethidium Bromide staining. Obtained picture was inverted and the band intensity was quantified by densitometry using QuantityOne software (BioRad). We determined PTBP1 mRNA expression levels with the TaqMan assay (Hs00259176-m1), which specifically measures the presence of exon 2. Ribosomal RNA (18S) (4308329) was used as an endogenous control to perform the comparative ΔC_T_ method. Both TaqMan assays were performed according to the manufacturer's suggested protocol using 10 ng of cDNA.

### Western Blot Analysis

Western blot analysis was performed as described previously [19]. Cleared supernatant fractions of protein lysates (20 µg) were loaded on 8% polyacrylamide gels, separated by electrophoresis and transferred on PVDF membranes. Membranes were blocked with 5% non-fat milk, incubated with primary antibodies for 1 h and with secondary horseradish peroxidase-conjugated antibodies (Sigma) in DPBS buffer for 45 min at room temperature. The signal was visualized by enhanced chemiluminescence (ECL Plus, Amersham). Densitometry analysis was performed using QuantityOne software (BioRad). The following primary antibodies were used: custom made rabbit polyclonal anti-α1 sGC antibodies raised against human C-terminal peptide FTPRSREELPPNFP (1∶1000 dilution); anti-β-actin (Sigma-Aldrich; 1∶7000 dilution); anti-PTBP1 (IMG-3559, IMGENEX, CA, 1∶2000 dilution); anti-HuR (3A2, Santa Cruz Biotechnology, 1∶200 dilution): pan-SR (1H4, Santa Cruz Biotechnology, 1∶200 dilution); anti-hnRNP A2/B1(G-16, Santa Cruz Biotechnology, 1∶200 dilution).

### H_2_O_2_ Cytotoxicity Assay

Cells grown on 96-well plates (1×10^5^ cells/ml) were treated with different concentrations of H_2_O_2_. After 24 hours, viable cells were visualized with trypan blue exclusion (Life Technologies/Invitrogen) to determine the cell number and viability by hemocytometer count. Knock-down of PTBP1 expression was performed using siRNA gene silencing (Santa Cruz Biotechnology) according to manufacturer's instructions.

### *In Silico* and Statistical Analysis

Calculation of relative splice site strengths was performed using the Human Splicing Finder version 2.4.1 online tool (www.umd.be/HSF/) [25], [26]. Mapping of hnRNP and SR regulatory sequences was performed using the ASD–Alternative Splicing/Splicing Rainbow tool from European Molecular Biology Laboratory (http://www.ebi.ac.uk/asd-srv/wb.cgi?method=8) [27]. The GUCY1A3 genomic sequences used for this analysis were directed from the human GRCh37/hg19 assembly and included: Exon 2 region (150 bp) chr4:156588521–156588670, Exon 4 region (568 bp) chr4:156617808–156618375) and Exon 5 region (150 bp) chr4:156624983–156625132. All data are presented as mean ± standard deviation. The Splicing Rainbow tabular output file was used to generate a predicted binding score based on the output score for individual nucleotide positions (see Table S1 for an example). Statistical comparisons between groups were performed by Student's t-test using Excel software with a p<0.05 considered statistically significant. Nonlinear regression and calculations of IC_50_ were performed using Graph Pad Prism 3.0 software (GraphPad Software).

## Supporting Information

Figure S1 **Distribution of Predicted Splicing Factor Binding Sites.** Shown is 294 bp of GUCY1A3 genomic sequence (GRCh37/hg19 assembly Chr4:156617821–156618114) spanning the intron 3 (low case)/exon 4 (upper case) junction. The position of alternative splice sites along with their predicted strength is shown. The C-α1 3′SS_1_ splice site is used to generate C-α1 mRNA isoform. Details regarding the use of other splice sites is reviewed in (Sharina, I.G., et al., *RNA splicing in regulation of nitric oxide receptor soluble guanylyl cyclase*. Nitric Oxide, 2011). The location of predicted regulatory sites for splicing factors examined in Figure 2B is shown. This information was derived using the ASD–Alternative Splicing/Splicing Rainbow tool with a detailed output of this analysis provided in Table S1.
(TIF)

Figure S2 **MDA453 cells response to H_2_O_2_ after siRNA-mediated knockdown of PTBP1.** **A**. *Cytotoxicity analysis*. MDA453 cells were plated on 24 well plates at 50% confluence in complete RPMI media. Next day, the transfection with siRNA has been performed according to manufacturer's recommendations (Santa Cruz Biotech., Inc). Cells were allowed to recover for 24 hours and treated with indicated concentrations of H2O2 in serum-free media. After 24 hours of incubation, the cells were lifted with Trypsin and total numbers of viable cells were counted with Vi-Cell XR Cell Viability Analyzer (Beckman Coulter). Data are shown as mean ±SD from three independent experiments. **B**. *Q-PCR analysis of PTBP1 mRNA levels in MDA453 cells transfected with scrambled control or PTBP1 siRNA*. At the time of cytotoxicity analysis, the cells were collected for RNA purification (RiboPure, Ambion) and Q-PCR analysis. Data are shown as mean ±SD from three independent experiments. PTB1 k/d – PTB1 knock down.
(PPTX)

Table S1 ASD Splicing Rainbow Output for GUCY1A3 Predicted Splicing Factor Binding Sites.
(DOC)
