# Supplementary figures and images for: Hydrogen Peroxide Alters Splicing of Soluble Guanylyl Cyclase and Selectively Modulates Expression of Splicing Regulators in Human Cancer Cells
Source: PLoS One. 2012 Jul 20;7(7):e41099. doi: 10.1371/journal.pone.0041099 (PMC3401163; doi:10.1371/journal.pone.0041099)

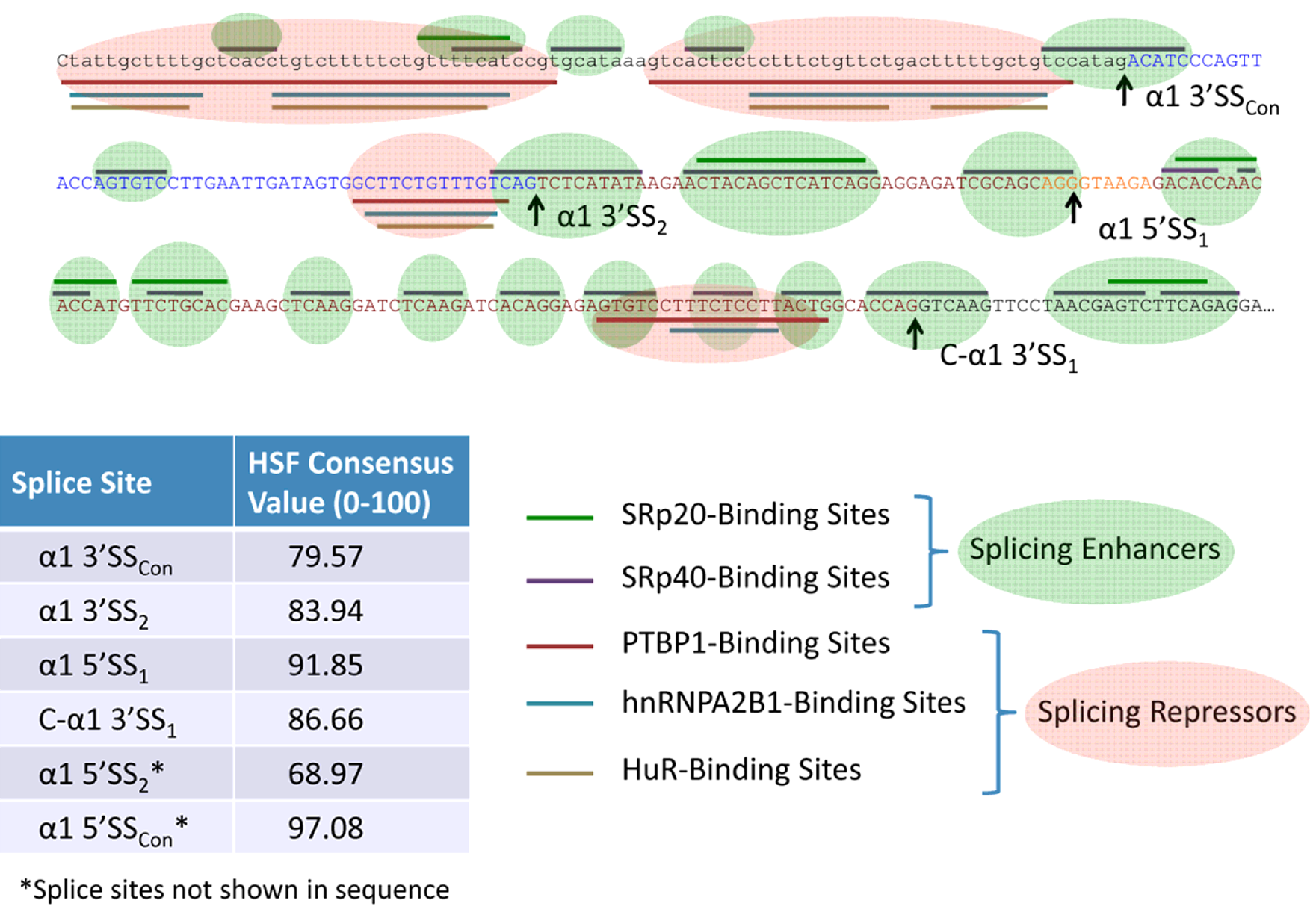

Supplement: Figure S1 — Distribution of Predicted Splicing Factor Binding Sites. Shown is 294 bp of GUCY1A3 genomic sequence (GRCh37/hg19 assembly Chr4:156617821–156618114) spanning the intron 3 (low case)/exon 4 (upper case) junction. The position of alternative splice sites along with their predicted strength is shown. The C-α1 3′SS1 splice site is used to generate C-α1 mRNA isoform. Details regarding the use of other splice sites is reviewed in (Sharina, I.G., et al., RNA splicing in regulation of nitric oxide receptor soluble guanylyl cyclase. Nitric Oxide, 2011). The location of predicted regulatory sites for splicing factors examined in Figure 2B is shown. This information was derived using the ASD–Alternative Splicing/Splicing Rainbow tool with a detailed output of this analysis provided in Table S1. (TIF) [file pone.0041099.s001.tif]

## Slide 1
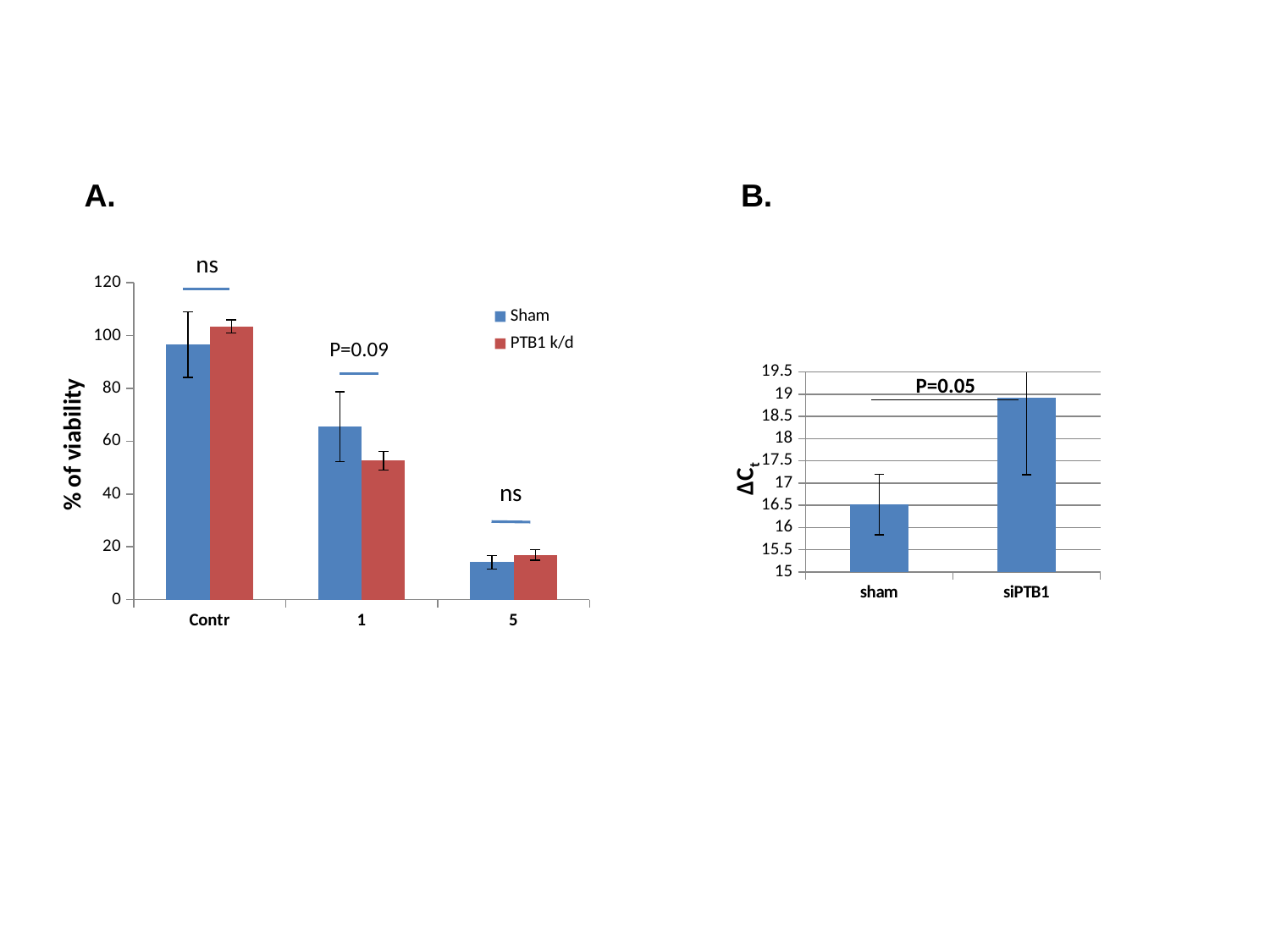

A.
B.
### Chart
| Category | av |
|---|---|
| sham | 16.513333333333332 |
| siPTB1 | 18.915 |ΔCt
P=0.05
ns
### Chart
| Category | Sham | PTB1 k/d |
|---|---|---|
| Contr | 96.49122807017544 | 103.50877192982456 |
| 1 | 65.49707602339181 | 52.63157894736843 |
| 5 | 14.15204678362573 | 16.959064327485383 |% of viability
P=0.09
ns

Supplement: Figure S2 — MDA453 cells response to H2O2 after siRNA-mediated knockdown of PTBP1. A. Cytotoxicity analysis. MDA453 cells were plated on 24 well plates at 50% confluence in complete RPMI media. Next day, the transfection with siRNA has been performed according to manufacturer's recommendations (Santa Cruz Biotech., Inc). Cells were allowed to recover for 24 hours and treated with indicated concentrations of H2O2 in serum-free media. After 24 hours of incubation, the cells were lifted with Trypsin and total numbers of viable cells were counted with Vi-Cell XR Cell Viability Analyzer (Beckman Coulter). Data are shown as mean ±SD from three independent experiments. B. Q-PCR analysis of PTBP1 mRNA levels in MDA453 cells transfected with scrambled control or PTBP1 siRNA. At the time of cytotoxicity analysis, the cells were collected for RNA purification (RiboPure, Ambion) and Q-PCR analysis. Data are shown as mean ±SD from three independent experiments. PTB1 k/d – PTB1 knock down. (PPTX) [file pone.0041099.s002.pptx]
