# Supplementary material for: Hydrogen Peroxide Alters Splicing of Soluble Guanylyl Cyclase and Selectively Modulates Expression of Splicing Regulators in Human Cancer Cells
Source: PLoS One. 2012 Jul 20;7(7):e41099. doi: 10.1371/journal.pone.0041099 (PMC3401163; doi:10.1371/journal.pone.0041099)
Supplement: Table S1 — ASD Splicing Rainbow Output for GUCY1A3 Predicted Splicing Factor Binding Sites. (DOC) [file pone.0041099.s003.doc]

**Supplemental Table 1. ASD Splicing Rainbow Output for GUCY1A3 Predicted Splicing Factor Binding Sites.**

| **Nuc_start** | **Nuc_end** | **Factor** | **Consensus_type** | **Score** |
| --- | --- | --- | --- | --- |
| 2 | 10 | hnRNP A2/B1 | poly-T | 6 T's (in 9) |
| 3 | 11 | hnRNP A2/B1 | poly-T | 6 T's (in 9) |
| 4 | 12 | hnRNP A2/B1 | poly-T | 6 T's (in 9) |
| 18 | 26 | hnRNP A2/B1 | poly-T | 6 T's (in 9) |
| 19 | 27 | hnRNP A2/B1 | poly-T | 7 T's (in 9) |
| 20 | 28 | hnRNP A2/B1 | poly-T | 6 T's (in 9) |
| 21 | 29 | hnRNP A2/B1 | poly-T | 7 T's (in 9) |
| 22 | 30 | hnRNP A2/B1 | poly-T | 6 T's (in 9) |
| 23 | 31 | hnRNP A2/B1 | poly-T | 7 T's (in 9) |
| 24 | 32 | hnRNP A2/B1 | poly-T | 7 T's (in 9) |
| 25 | 33 | hnRNP A2/B1 | poly-T | 7 T's (in 9) |
| 26 | 34 | hnRNP A2/B1 | poly-T | 7 T's (in 9) |
| 27 | 35 | hnRNP A2/B1 | poly-T | 6 T's (in 9) |
| 29 | 37 | hnRNP A2/B1 | poly-T | 6 T's (in 9) |
| 57 | 65 | hnRNP A2/B1 | poly-T | 6 T's (in 9) |
| 58 | 66 | hnRNP A2/B1 | poly-T | 6 T's (in 9) |
| 59 | 67 | hnRNP A2/B1 | poly-T | 6 T's (in 9) |
| 60 | 68 | hnRNP A2/B1 | poly-T | 6 T's (in 9) |
| 68 | 76 | hnRNP A2/B1 | poly-T | 6 T's (in 9) |
| 71 | 79 | hnRNP A2/B1 | poly-T | 6 T's (in 9) |
| 72 | 80 | hnRNP A2/B1 | poly-T | 6 T's (in 9) |
| 73 | 81 | hnRNP A2/B1 | poly-T | 6 T's (in 9) |
| 124 | 132 | hnRNP A2/B1 | poly-T | 6 T's (in 9) |
| 125 | 133 | hnRNP A2/B1 | poly-T | 6 T's (in 9) |
| 126 | 134 | hnRNP A2/B1 | poly-T | 6 T's (in 9) |
| 247 | 255 | hnRNP A2/B1 | poly-T | 6 T's (in 9) |
| 129 | 132 | hnRNP A2/B1 | GTTTG | Exact match |
| 23 | 27 | hnRNP C1/C2 | TTTTT | Exact match |
| 72 | 76 | hnRNP C1/C2 | TTTTT | Exact match |
| 1 | 10 | hnRNP I (PTB) | poly-Y | 8 pyrimidines (in 10) |
| 2 | 11 | hnRNP I (PTB) | poly-Y | 8 pyrimidines (in 10) |
| 4 | 13 | hnRNP I (PTB) | poly-Y | 8 pyrimidines (in 10) |
| 5 | 14 | hnRNP I (PTB) | poly-Y | 8 pyrimidines (in 10) |
| 6 | 15 | hnRNP I (PTB) | poly-Y | 8 pyrimidines (in 10) |
| 7 | 16 | hnRNP I (PTB) | poly-Y | 8 pyrimidines (in 10) |
| 8 | 17 | hnRNP I (PTB) | poly-Y | 8 pyrimidines (in 10) |
| 9 | 18 | hnRNP I (PTB) | poly-Y | 8 pyrimidines (in 10) |
| 10 | 19 | hnRNP I (PTB) | poly-Y | 8 pyrimidines (in 10) |
| 13 | 22 | hnRNP I (PTB) | poly-Y | 8 pyrimidines (in 10) |
| 14 | 23 | hnRNP I (PTB) | poly-Y | 8 pyrimidines (in 10) |
| 15 | 24 | hnRNP I (PTB) | poly-Y | 8 pyrimidines (in 10) |
| 16 | 25 | hnRNP I (PTB) | poly-Y | 8 pyrimidines (in 10) |
| 17 | 26 | hnRNP I (PTB) | poly-Y | 9 pyrimidines (in 10) |
| 18 | 27 | hnRNP I (PTB) | poly-Y | 9 pyrimidines (in 10) |
| 19 | 28 | hnRNP I (PTB) | poly-Y | 9 pyrimidines (in 10) |
| 20 | 29 | hnRNP I (PTB) | poly-Y | 9 pyrimidines (in 10) |
| 21 | 30 | hnRNP I (PTB) | poly-Y | 9 pyrimidines (in 10) |
| 22 | 31 | hnRNP I (PTB) | poly-Y | 9 pyrimidines (in 10) |
| 23 | 32 | hnRNP I (PTB) | poly-Y | 9 pyrimidines (in 10) |
| 24 | 33 | hnRNP I (PTB) | poly-Y | 9 pyrimidines (in 10) |
| 25 | 34 | hnRNP I (PTB) | poly-Y | 9 pyrimidines (in 10) |
| 26 | 35 | hnRNP I (PTB) | poly-Y | 9 pyrimidines (in 10) |
| 27 | 36 | hnRNP I (PTB) | poly-Y | 8 pyrimidines (in 10) |
| 28 | 37 | hnRNP I (PTB) | poly-Y | 8 pyrimidines (in 10) |
| 29 | 38 | hnRNP I (PTB) | poly-Y | 8 pyrimidines (in 10) |
| 30 | 39 | hnRNP I (PTB) | poly-Y | 8 pyrimidines (in 10) |
| 31 | 40 | hnRNP I (PTB) | poly-Y | 8 pyrimidines (in 10) |
| 32 | 41 | hnRNP I (PTB) | poly-Y | 8 pyrimidines (in 10) |
| 49 | 58 | hnRNP I (PTB) | poly-Y | 8 pyrimidines (in 10) |
| 50 | 59 | hnRNP I (PTB) | poly-Y | 9 pyrimidines (in 10) |
| 51 | 60 | hnRNP I (PTB) | poly-Y | 9 pyrimidines (in 10) |
| 52 | 61 | hnRNP I (PTB) | poly-Y | 9 pyrimidines (in 10) |
| 53 | 62 | hnRNP I (PTB) | poly-Y | 10 pyrimidines (in 10) |
| 54 | 63 | hnRNP I (PTB) | poly-Y | 10 pyrimidines (in 10) |
| 55 | 64 | hnRNP I (PTB) | poly-Y | 9 pyrimidines (in 10) |
| 56 | 65 | hnRNP I (PTB) | poly-Y | 9 pyrimidines (in 10) |
| 57 | 66 | hnRNP I (PTB) | poly-Y | 9 pyrimidines (in 10) |
| 58 | 67 | hnRNP I (PTB) | poly-Y | 9 pyrimidines (in 10) |
| 59 | 68 | hnRNP I (PTB) | poly-Y | 9 pyrimidines (in 10) |
| 60 | 69 | hnRNP I (PTB) | poly-Y | 8 pyrimidines (in 10) |
| 65 | 74 | hnRNP I (PTB) | poly-Y | 8 pyrimidines (in 10) |
| 66 | 75 | hnRNP I (PTB) | poly-Y | 8 pyrimidines (in 10) |
| 67 | 76 | hnRNP I (PTB) | poly-Y | 8 pyrimidines (in 10) |
| 70 | 79 | hnRNP I (PTB) | poly-Y | 8 pyrimidines (in 10) |
| 71 | 80 | hnRNP I (PTB) | poly-Y | 8 pyrimidines (in 10) |
| 72 | 81 | hnRNP I (PTB) | poly-Y | 8 pyrimidines (in 10) |
| 73 | 82 | hnRNP I (PTB) | poly-Y | 8 pyrimidines (in 10) |
| 74 | 83 | hnRNP I (PTB) | poly-Y | 8 pyrimidines (in 10) |
| 123 | 132 | hnRNP I (PTB) | poly-Y | 8 pyrimidines (in 10) |
| 124 | 133 | hnRNP I (PTB) | poly-Y | 8 pyrimidines (in 10) |
| 125 | 134 | hnRNP I (PTB) | poly-Y | 8 pyrimidines (in 10) |
| 126 | 135 | hnRNP I (PTB) | poly-Y | 8 pyrimidines (in 10) |
| 241 | 250 | hnRNP I (PTB) | poly-Y | 8 pyrimidines (in 10) |
| 242 | 251 | hnRNP I (PTB) | poly-Y | 9 pyrimidines (in 10) |
| 243 | 252 | hnRNP I (PTB) | poly-Y | 9 pyrimidines (in 10) |
| 244 | 253 | hnRNP I (PTB) | poly-Y | 10 pyrimidines (in 10) |
| 245 | 254 | hnRNP I (PTB) | poly-Y | 10 pyrimidines (in 10) |
| 246 | 255 | hnRNP I (PTB) | poly-Y | 10 pyrimidines (in 10) |
| 247 | 256 | hnRNP I (PTB) | poly-Y | 9 pyrimidines (in 10) |
| 248 | 257 | hnRNP I (PTB) | poly-Y | 9 pyrimidines (in 10) |
| 249 | 258 | hnRNP I (PTB) | poly-Y | 9 pyrimidines (in 10) |
| 250 | 259 | hnRNP I (PTB) | poly-Y | 8 pyrimidines (in 10) |
| 21 | 24 | hnRNP I (PTB) | TCTT | Exact match |
| 57 | 60 | hnRNP I (PTB) | TCTT | Exact match |
| 284 | 287 | hnRNP I (PTB) | TCTT | Exact match |
| 81 | 86 | hnRNP K | matrix 6-mer | 4.179 |
| 91 | 96 | hnRNP K | matrix 6-mer | 5.39 |
| 223 | 228 | hnRNP K | matrix 6-mer | 4.256 |
| 251 | 256 | hnRNP K | matrix 6-mer | 4.179 |
| 273 | 278 | hnRNP K | matrix 6-mer | 4.179 |
| 274 | 279 | hnRNP K | matrix 6-mer | 4.256 |
| 163 | 172 | hnRNP U | poly-G | 7.5 G's (in 10) |
| 179 | 188 | hnRNP U | poly-G | 7.5 G's (in 10) |
| 180 | 189 | hnRNP U | poly-G | 7.5 G's (in 10) |
| 235 | 244 | hnRNP U | poly-G | 7.5 G's (in 10) |
| 2 | 11 | HuR | poly-T | 7 T's (in 10) |
| 18 | 27 | HuR | poly-T | 7 T's (in 10) |
| 19 | 28 | HuR | poly-T | 7 T's (in 10) |
| 20 | 29 | HuR | poly-T | 7 T's (in 10) |
| 21 | 30 | HuR | poly-T | 7 T's (in 10) |
| 22 | 31 | HuR | poly-T | 7 T's (in 10) |
| 23 | 32 | HuR | poly-T | 8 T's (in 10) |
| 24 | 33 | HuR | poly-T | 8 T's (in 10) |
| 25 | 34 | HuR | poly-T | 8 T's (in 10) |
| 26 | 35 | HuR | poly-T | 7 T's (in 10) |
| 57 | 66 | HuR | poly-T | 7 T's (in 10) |
| 59 | 68 | HuR | poly-T | 7 T's (in 10) |
| 72 | 81 | HuR | poly-T | 7 T's (in 10) |
| 125 | 134 | HuR | poly-T | 7 T's (in 10) |
| 23 | 27 | HuR | (T/A)TTT(T/A) | Exact match |
| 72 | 76 | HuR | (T/A)TTT(T/A) | Exact match |
| 184 | 192 | 9G8 | matrix 9-mer | 6.664 |
| 52 | 62 | SRp20 | matrix 11-mer | 9 |
| 30 | 37 | SRp20 | matrix 8-mer | 5.217 |
| 157 | 164 | SRp20 | matrix 8-mer | 5.783 |
| 194 | 201 | SRp20 | matrix 8-mer | 4.838 |
| 203 | 210 | SRp20 | matrix 8-mer | 5.054 |
| 283 | 290 | SRp20 | matrix 8-mer | 6.119 |
| 151 | 157 | SRp20 | matrix 7-mer | 6.522 |
| 190 | 196 | SRp20 | matrix 7-mer | 5.364 |
| 33 | 38 | SRp40 | matrix 6-mer | 4.165 |
| 41 | 46 | SRp40 | matrix 6-mer | 5.683 |
| 81 | 86 | SRp40 | matrix 6-mer | 3.568 |
| 87 | 92 | SRp40 | matrix 6-mer | 3.512 |
| 102 | 107 | SRp40 | matrix 6-mer | 2.658 |
| 139 | 144 | SRp40 | matrix 6-mer | 2.826 |
| 141 | 146 | SRp40 | matrix 6-mer | 3.161 |
| 152 | 157 | SRp40 | matrix 6-mer | 4.791 |
| 157 | 162 | SRp40 | matrix 6-mer | 3.422 |
| 173 | 178 | SRp40 | matrix 6-mer | 4.046 |
| 240 | 245 | SRp40 | matrix 6-mer | 2.658 |
| 280 | 285 | SRp40 | matrix 6-mer | 3.35 |
| 14 | 18 | SRp40 | matrix 5-mer | 2.592 |
| 34 | 38 | SRp40 | matrix 5-mer | 2.214 |
| 52 | 56 | SRp40 | matrix 5-mer | 3.359 |
| 88 | 92 | SRp40 | matrix 5-mer | 3.564 |
| 134 | 138 | SRp40 | matrix 5-mer | 1.822 |
| 150 | 154 | SRp40 | matrix 5-mer | 2.981 |
| 153 | 157 | SRp40 | matrix 5-mer | 5.593 |
| 158 | 162 | SRp40 | matrix 5-mer | 2.214 |
| 161 | 165 | SRp40 | matrix 5-mer | 3.824 |
| 174 | 178 | SRp40 | matrix 5-mer | 3.287 |
| 177 | 181 | SRp40 | matrix 5-mer | 2.868 |
| 189 | 193 | SRp40 | matrix 5-mer | 3.941 |
| 195 | 199 | SRp40 | matrix 5-mer | 3.941 |
| 204 | 208 | SRp40 | matrix 5-mer | 3.66 |
| 216 | 220 | SRp40 | matrix 5-mer | 1.796 |
| 225 | 229 | SRp40 | matrix 5-mer | 1.796 |
| 233 | 237 | SRp40 | matrix 5-mer | 5.174 |
| 249 | 253 | SRp40 | matrix 5-mer | 2.009 |
| 256 | 260 | SRp40 | matrix 5-mer | 4.591 |
| 263 | 267 | SRp40 | matrix 5-mer | 2.254 |
| 268 | 272 | SRp40 | matrix 5-mer | 1.796 |
| 279 | 283 | SRp40 | matrix 5-mer | 2.112 |
| 287 | 291 | SRp40 | matrix 5-mer | 1.822 |
| 289 | 293 | SRp40 | matrix 5-mer | 1.976 |
| 1 | 7 | SRp55 | matrix 7-mer | 2.709 |
| 12 | 18 | SRp55 | matrix 7-mer | 5.706 |
| 14 | 20 | SRp55 | matrix 7-mer | 2.971 |
| 25 | 31 | SRp55 | matrix 7-mer | 2.062 |
| 32 | 38 | SRp55 | matrix 7-mer | 2.866 |
| 34 | 40 | SRp55 | matrix 7-mer | 2.618 |
| 50 | 56 | SRp55 | matrix 7-mer | 6.417 |
| 59 | 65 | SRp55 | matrix 7-mer | 2.062 |
| 64 | 70 | SRp55 | matrix 7-mer | 2.229 |
| 77 | 83 | SRp55 | matrix 7-mer | 2.976 |
| 86 | 92 | SRp55 | matrix 7-mer | 2.002 |
| 97 | 103 | SRp55 | matrix 7-mer | 4.181 |
| 117 | 123 | SRp55 | matrix 7-mer | 2.007 |
| 142 | 148 | SRp55 | matrix 7-mer | 2.258 |
| 148 | 154 | SRp55 | matrix 7-mer | 3.567 |
| 151 | 157 | SRp55 | matrix 7-mer | 5.567 |
| 156 | 162 | SRp55 | matrix 7-mer | 3.54 |
| 158 | 164 | SRp55 | matrix 7-mer | 2.17 |
| 159 | 165 | SRp55 | matrix 7-mer | 2.307 |
| 172 | 178 | SRp55 | matrix 7-mer | 3.594 |
| 174 | 180 | SRp55 | matrix 7-mer | 2.465 |
| 187 | 193 | SRp55 | matrix 7-mer | 4.168 |
| 192 | 198 | SRp55 | matrix 7-mer | 2.736 |
| 193 | 199 | SRp55 | matrix 7-mer | 3.996 |
| 202 | 208 | SRp55 | matrix 7-mer | 4.37 |
| 205 | 211 | SRp55 | matrix 7-mer | 3.401 |
| 214 | 220 | SRp55 | matrix 7-mer | 5.275 |
| 223 | 229 | SRp55 | matrix 7-mer | 5.108 |
| 231 | 237 | SRp55 | matrix 7-mer | 6.286 |
| 247 | 253 | SRp55 | matrix 7-mer | 4.692 |
| 254 | 260 | SRp55 | matrix 7-mer | 5.439 |
| 258 | 264 | SRp55 | matrix 7-mer | 2.537 |
| 260 | 266 | SRp55 | matrix 7-mer | 4.855 |
| 266 | 272 | SRp55 | matrix 7-mer | 3.729 |
| 275 | 281 | SRp55 | matrix 7-mer | 3.623 |
| 285 | 291 | SRp55 | matrix 7-mer | 2.208 |
| 287 | 293 | SRp55 | matrix 7-mer | 3.897 |
| 37 | 42 | SRp55 | matrix 6-mer | 4.432 |
| 41 | 46 | SRp55 | matrix 6-mer | 2.677 |
| 81 | 86 | SRp55 | matrix 6-mer | 3.28 |
| 134 | 139 | SRp55 | matrix 6-mer | 2.287 |
| 208 | 213 | SRp55 | matrix 6-mer | 2.41 |
| 251 | 256 | SRp55 | matrix 6-mer | 3.28 |
| 273 | 278 | SRp55 | matrix 6-mer | 2.059 |
| 143 | 150 | Tra2B | AAGAAGAA | 6 matches |
| 146 | 153 | Tra2B | AAGAAGAA | 6 matches |
| 162 | 169 | Tra2B | AAGAAGAA | 6 matches |
| 163 | 170 | Tra2B | AAGAAGAA | 6.5 matches |
| 165 | 172 | Tra2B | AAGAAGAA | 6 matches |
| 184 | 191 | Tra2B | AAGAAGAA | 6 matches |
| 234 | 241 | Tra2B | AAGAAGAA | 6 matches |
| 166 | 175 | ASF/SF2 | matrix 10-mer | 4.489 |
| 184 | 193 | ASF/SF2 | matrix 10-mer | 3.977 |
| 206 | 215 | ASF/SF2 | matrix 10-mer | 6.812 |
| 235 | 244 | ASF/SF2 | matrix 10-mer | 3.537 |
| 144 | 152 | ASF/SF2 | matrix 9-mer | 5.362 |
| 147 | 155 | ASF/SF2 | matrix 9-mer | 3.452 |
| 163 | 171 | ASF/SF2 | matrix 9-mer | 2.849 |
| 166 | 174 | ASF/SF2 | matrix 9-mer | 3.543 |
| 235 | 243 | ASF/SF2 | matrix 9-mer | 2.941 |
| 13 | 19 | ASF/SF2 | matrix 7-mer | 2.809 |
| 15 | 21 | ASF/SF2 | matrix 7-mer | 2.623 |
| 35 | 41 | ASF/SF2 | matrix 7-mer | 2.821 |
| 67 | 73 | ASF/SF2 | matrix 7-mer | 3.238 |
| 160 | 166 | ASF/SF2 | matrix 7-mer | 2.669 |
| 163 | 169 | ASF/SF2 | matrix 7-mer | 2.787 |
| 173 | 179 | ASF/SF2 | matrix 7-mer | 3.183 |
| 188 | 194 | ASF/SF2 | matrix 7-mer | 2.78 |
| 206 | 212 | ASF/SF2 | matrix 7-mer | 3.336 |
| 215 | 221 | ASF/SF2 | matrix 7-mer | 2.426 |
| 224 | 230 | ASF/SF2 | matrix 7-mer | 3.366 |
| 232 | 238 | ASF/SF2 | matrix 7-mer | 6.378 |
| 261 | 267 | ASF/SF2 | matrix 7-mer | 2.271 |
| 276 | 282 | ASF/SF2 | matrix 7-mer | 3.563 |
| 288 | 294 | ASF/SF2 | matrix 7-mer | 6.058 |
| 89 | 99 | SC35 | matrix 11-mer B | 6.669 |
| 176 | 184 | SC35 | matrix 9-mer F | 7.652 |
| 235 | 243 | SC35 | matrix 9-mer F | 9.295 |
| 5 | 11 | SC35 | matrix 7-mer G | 6.615 |
| 26 | 32 | SC35 | matrix 7-mer G | 6.615 |
| 60 | 66 | SC35 | matrix 7-mer G | 6.615 |
| 76 | 82 | SC35 | matrix 7-mer G | 8.562 |
| 125 | 131 | SC35 | matrix 7-mer G | 6.615 |
| 163 | 169 | SC35 | matrix 7-mer H | 4.385 |
| 166 | 172 | SC35 | matrix 7-mer H | 9.022 |
| 219 | 225 | SC35 | matrix 7-mer H | 4.7 |
| 235 | 241 | SC35 | matrix 7-mer H | 7.215 |
| 35 | 42 | SC35 | matrix 8-mer I | 2.585 |
| 80 | 87 | SC35 | matrix 8-mer I | 2.538 |
| 89 | 96 | SC35 | matrix 8-mer I | 2.716 |
| 95 | 102 | SC35 | matrix 8-mer I | 2.344 |
| 98 | 105 | SC35 | matrix 8-mer I | 2.531 |
| 122 | 129 | SC35 | matrix 8-mer I | 5.346 |
| 137 | 144 | SC35 | matrix 8-mer I | 3.465 |
| 149 | 156 | SC35 | matrix 8-mer I | 2.531 |
| 170 | 177 | SC35 | matrix 8-mer I | 3.898 |
| 187 | 194 | SC35 | matrix 8-mer I | 2.377 |
| 188 | 195 | SC35 | matrix 8-mer I | 2.115 |
| 202 | 209 | SC35 | matrix 8-mer I | 2.661 |
| 220 | 227 | SC35 | matrix 8-mer I | 2.451 |
| 221 | 228 | SC35 | matrix 8-mer I | 3.498 |
| 229 | 236 | SC35 | matrix 8-mer I | 4.093 |
| 259 | 266 | SC35 | matrix 8-mer I | 2.882 |
| 271 | 278 | SC35 | matrix 8-mer I | 3.367 |
| 272 | 279 | SC35 | matrix 8-mer I | 2.128 |
| 283 | 290 | SC35 | matrix 8-mer I | 3.783 |
| 7 | 10 | U2AF65 | poly-Y | CTTT |
| 8 | 11 | U2AF65 | poly-Y | TTTT |
| 21 | 24 | U2AF65 | poly-Y | TCTT |
| 22 | 25 | U2AF65 | poly-Y | CTTT |
| 23 | 26 | U2AF65 | poly-Y | TTTT |
| 24 | 27 | U2AF65 | poly-Y | TTTT |
| 25 | 28 | U2AF65 | poly-Y | TTTC |
| 26 | 29 | U2AF65 | poly-Y | TTCT |
| 31 | 34 | U2AF65 | poly-Y | TTTT |
| 32 | 35 | U2AF65 | poly-Y | TTTC |
| 57 | 60 | U2AF65 | poly-Y | TCTT |
| 58 | 61 | U2AF65 | poly-Y | CTTT |
| 59 | 62 | U2AF65 | poly-Y | TTTC |
| 60 | 63 | U2AF65 | poly-Y | TTCT |
| 65 | 68 | U2AF65 | poly-Y | TTCT |
| 71 | 74 | U2AF65 | poly-Y | CTTT |
| 72 | 75 | U2AF65 | poly-Y | TTTT |
| 73 | 76 | U2AF65 | poly-Y | TTTT |
| 125 | 128 | U2AF65 | poly-Y | TTCT |
| 203 | 206 | U2AF65 | poly-Y | TTCT |
| 246 | 249 | U2AF65 | poly-Y | CTTT |
| 247 | 250 | U2AF65 | poly-Y | TTTC |
| 248 | 251 | U2AF65 | poly-Y | TTCT |
| 284 | 287 | U2AF65 | poly-Y | TCTT |
| 58 | 75 | Sxl | matrix 18-mer | 11.27 |
